# Pathophysiologic Mechanisms of Cardiovascular Disease in Patients With Bipolar Disorder

**DOI:** 10.7759/cureus.93029

**Published:** 2025-09-23

**Authors:** Dhiya Ram, Sukriti Prashar, Niraj Pathak, Shahzaib Chughtai, Stephanie Nagy, Raymond L Ownby, Marc M Kesselman

**Affiliations:** 1 Internal Medicine, Nova Southeastern University Dr. Kiran C. Patel College of Osteopathic Medicine, Fort Lauderdale, USA; 2 Internal Medicine, Central Michigan University College of Medicine, Saginaw, USA; 3 Rheumatology, Nova Southeastern University Dr. Kiran C. Patel College of Osteopathic Medicine, Fort Lauderdale, USA; 4 Psychiatry and Behavioral Sciences, Nova Southeastern University Dr. Kiran C. Patel College of Osteopathic Medicine, Fort Lauderdale, USA; 5 Rheumatology, Kiran C. Patel College of Osteopathic Medicine, Nova Southeastern University, Davie, USA

**Keywords:** bipolar disorder, cardiovascular disease, inflammation, pathophysiology, psychocardiology

## Abstract

Bipolar disorder (BD) is a mental health condition characterized by periodic intense emotional states ranging between mania and depression. It has a strong association with comorbid cardiovascular disease (CVD), which contributes greatly to the mortality among this patient population. While a part of this association can be attributed to antipsychotic medication use, this systematic review highlights and offers a comprehensive analysis of other pathophysiological mechanisms that drive CVD in BD patients beyond pharmacologic and psychosocial factors. Through a systematic search of EMBASE, OVID, and Web of Science databases, a total of 282 full articles were screened, with 31 total studies being included. To avoid bias, four authors reviewed these articles and discussed conflicts until a consensus was reached. This systematic review thus synthesized the data into five broad categories of pathophysiological mechanisms: genetics, inflammatory markers, endothelial dysfunction, oxidative stress, and structural cardiovascular changes that contribute to CVD in the BD population. A further study into how these mechanisms are integrated in the BD population showed that there were 129 shared loci between BD and CVD, along with specific gene polymorphisms that are associated with cardiomyopathy and arrhythmias. Furthermore, elevated levels of inflammatory markers in BD patients contribute to atherogenesis via oxidative stress. In turn, increased atherogenesis leads to elevations in coronary calcium and left ventricular mass index, especially in male patients with BD. The elevations in inflammatory markers and changes in endothelial function appear to be associated with mood lability in BD patients. These findings further cement how fundamentally BD as an inflammatory condition contributes to the CVD burden in its population. This paper thus suggests proactive measures to be implemented in healthcare settings to assess the multiple pathophysiologic mechanisms responsible for the increased risk of CVD in BD patients. Further research in the realm of genetic biomarkers is vital to explicate the pathogenesis of CVD in BD patients.

## Introduction and background

Bipolar disorder (BD) is a chronic psychiatric illness characterized by alternating episodes of mania and depression, affecting approximately 1-2% of the global population [1]. Beyond mood instability, BD is increasingly recognized for its association with significant medical comorbidities; most notably, cardiovascular disease (CVD), the leading cause of premature mortality among individuals with BD [2,3].

BD is linked to an elevated and earlier onset of CVD, with evidence of both clinical and subclinical accelerated atherosclerosis [4]. BD patients have been noted to have a higher risk of myocardial infarction and stroke, but are less likely to receive interventional cardiovascular care [5]. In fact, physical conditions such as CVD account for 70% of deaths in BD patients [5]. Thus, the association between BD and CVD appears to be as strong as the association between major depressive disorder (MDD) and CVD, but remains less commonly recognized [4].

While lifestyle factors contribute to this increased CVD risk, accumulating evidence points to intrinsic biological and pathophysiological links between BD and cardiovascular dysfunction [6]. These include autonomic dysregulation - such as altered heart rate variability and baroreflex sensitivity [7] - as well as chronic low-grade inflammation, with elevated cytokines like IL-6 and TNF-α promoting vascular dysfunction [4,8]. Oxidative and nitrosative stress, commonly seen in BD, contribute to both mood instability and vascular injury [9], while mitochondrial dysfunction may impair cerebral and vascular energy metabolism [10]. Moreover, lipid abnormalities - elevated LDL and triglycerides and reduced HDL - further amplify CVD risk [11].

This systematic review aims to synthesize current evidence on the pathophysiological mechanisms linking BD to cardiovascular disease, with particular attention to autonomic, inflammatory, metabolic, and oxidative pathways that may underlie this comorbidity.

## Review

Methods

**Figure 1:**
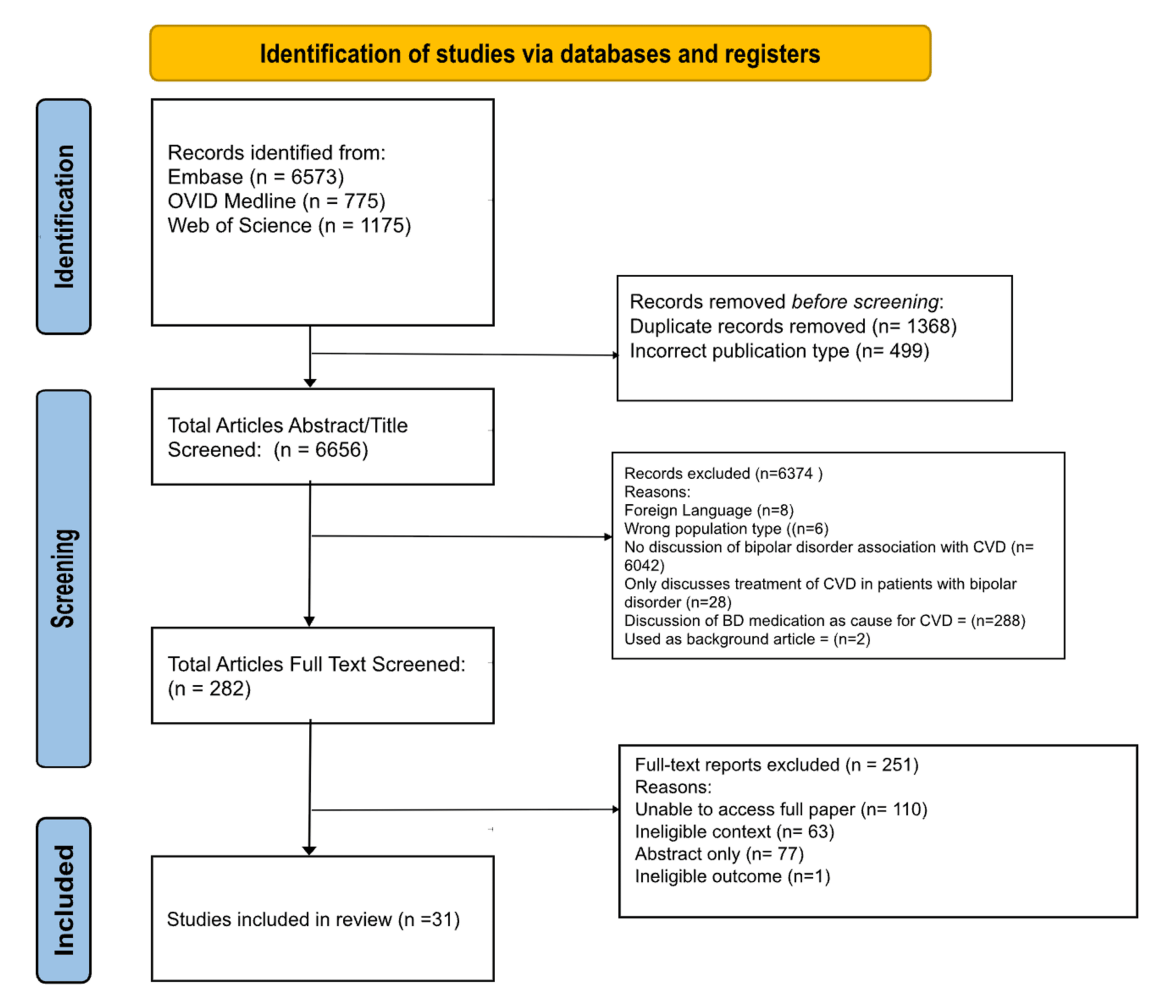
PRISMA flowchart of the selection procedure PRISMA: Preferred Reporting Items for Systematic Reviews and Meta-Analyses, BD: bipolar disorder, CVD: cardiovascular disease Source: [12]

An initial literature search was conducted on Google Scholar to gain a brief overview of the topic, after which certain keywords were sought from relevant articles. With BD being the pathology of interest, the keyword “bipolar disorder” was searched across abstracts, keywords, and titles, alongside second-level keywords including “bipolar affective disorder” OR “bipolar and related disorders” OR “bipolar illness” OR “bipolar psychosis” OR “depression, manic” OR “manic depression” OR “manic depression psychosis” OR “manic depressive” OR “manic depressive disease” OR “manic depressive disorder” OR “manic depressive illness” OR “manic depressive psychosis” OR “manic depressive reaction” OR “manic depressive syndrome” OR “maniodepressive psychosis” OR “mano depressive syndrome” OR “psychosis, manic depressive” OR “bipolar disorder”. Next keywords such as “cardiovascular disease” OR “angiocardiopathy” OR “angiocardiovascular disease” OR “cardiovascular complication” OR “cardiovascular diseases” OR “cardiovascular disorder” OR “cardiovascular disturbance” OR “cardiovascular lesion” OR “cardiovascular syndrome” OR “cardiovascular vegetative disorder” OR “complication, cardiovascular” OR “disease, cardiovascular” OR “major adverse cardiovascular event” OR “cardiovascular disease”. Articles from these two searches were connected via the Boolean operator AND. The search strategy was replicated across three databases, namely, EMBASE, Ovid MEDLINE, and Web of Science, on October 13, 2024. The initial search yielded 8,523 articles. Of note, the authors of this systematic narrative review initially intended to study all aspects of the relationship between BD and CVD, including but not limited to pathophysiologic mechanisms, risk factors, population trends, and treatment modalities. However, due to the high amount of literature yielded from the initial search, the authors chose to focus this systematic narrative review on the intrinsic pathophysiologic mechanisms of CVD in BD patients. Before screening and the article selection process, EndNote 21 was used to remove duplicates, after which 1,368 articles were imported to Rayyan. Rayyan was used to organize articles during the Tier 1 and Tier 2 screening stages. Three contributing authors independently reviewed each article’s title, abstract, and full text, depending on the stage of review, to determine inclusion or exclusion based on predefined eligibility criteria. Based on this approach, 499 articles were immediately excluded for being incorrect publication types for systematic reviews. The remaining 6,656 articles went through Tier 1 title and abstract screening, after which, 6,374 articles were excluded, leaving 282 articles. The eligibility criteria during the Tier 1 stage were designed to investigate the intrinsic pathophysiologic mechanisms linking BD and CVD. As a result, articles were excluded for the following reasons: if they were in a foreign non-English language, assessed the wrong population type, did not discuss pathophysiologic relationship between BD and CVD, focused only on treatment of CVD in BD patients, discussed BD pharmacologic treatment as the cause of CVD development, and was used as a background article due to lack of review-able data. Of note, it is important to recognize that the studies that primarily attributed cardiovascular risk to the effects of pharmacological treatments for BD were excluded during the Tier 1 screening stage. This decision was made to isolate non-iatrogenic mechanisms and better characterize the underlying intrinsic predisposition to CVD observed in BD populations. Two hundred fifty-one more articles were excluded after full-text screening at the Tier 2 stage, leaving 31 articles to be included in the systematic review. Articles included finally met the final inclusion criteria of the ability of authors to access and assess the full scientific paper, an eligible context in terms of pathophysiology of CVD in BD patients, and an eligible outcome. The PRISMA flowchart (Preferred Reporting Items for Systematic Reviews and Meta-Analyses) details the study’s article screening and selection process (Figure 1) [12] . 

Data were extrapolated from the final 31 papers and charted independently by each of the four reviewers onto an Excel data extraction form (Microsoft Corp., USA). The variables utilized in the form included aspects of study design, methodology, and outcomes. These variables were author, year, title, journal, study design, population studied, major findings, and relevant remaining gaps. Artificial intelligence or any other form of automated assistance was not utilized at any stage of this review. All processes, including screening, selection, and analysis, were conducted manually by the authors of this review.

Results

Data were extrapolated from the final 31 papers and charted independently by each of the three reviewers. The variables utilized in the form included year of publication, aspects of the study design, pathophysiologic mechanism of CVD explored in BD patients, and the number of subjects. Table 1 shows the 31 articles analyzed for the publication year, study design, pathophysiologic mechanism, and number of subjects included in selected studies.

**Table 1:** Analysis of selected studies Source: [13-43]

Study	Year	Study design	Pathophysiologic mechanism	Number of subjects
Chen et al. [13]	2020	Cohort study	Cardiovascular dysregulation	46,490
Prieto et al. [14]	2016	Cohort study	Cardiovascular dysregulation	668
McGowan et al. [15]	2021	Observational study	Cardiovascular dysregulation	106
Najar et al. [16]	2022	Cohort study	Cardiovascular dysregulation	395
Wageck et al. [17]	2018	Cross-sectional study	Cardiovascular dysregulation	41
Chen et al. [18]	2024	Case-control study	Cardiovascular dysregulation	104 (52 case + 52 control)
Nunes et al. [19]	2015	Case-control study	Cardiovascular dysregulation	331 (134 case + 197 control)
Knedeisen et al. [20]	2023	Cross-sectional study	Cardiovascular dysregulation	54
Goldstein et al. [21]	2022	Randomized control trial	Endothelial dysfunction	86
Kennedy et al. [22]	2022	Randomized control trial	Endothelial dysfunction	209
Liou et al. [23]	2022	Cross-sectional study	Endothelial dysfunction	98
Kennedy et al. [24]	2023	Case-control study	Endothelial dysfunction	115 (66 case + 49 control)
Barbosa et al. [25]	2017	Cross-sectional	Endothelial dysfunction	70
Stubbs et al. [26]	2015	Cross-sectional study	Inflammation	250
Prins et al. [27]	2016	Cross-sectional study	Inflammation	123,865
Oral et al. [28]	2019	Cross-sectional study	Inflammation	88
Turan et al. [29]	2014	Case-control study	Inflammation	100 (50 case + 50 control)
Tsai et al. [30]	2023	Cross-sectional study	Inflammation	110
Tsai et al. [31]	2017	Case-control study	Inflammation	5416
Yang et al. [32]	2018	Case-control study	Inflammation	79 (46 case + 33 control)
Rødevand et al. [33]	2021	Genetic association study	Inflammation	-
Hatch et al. [34]	2015	Cohort study	Oxidative stress and metabolic dysregulation	30
Coello et al. [35]	2024	Case-control study	Oxidative stress and metabolic dysregulation	273 (133 case + 57 unaffected relatives + 83 control)
Xie et al. [36]	2023	Cross-sectional study	Oxidative stress and metabolic dysregulation	791
Fürtjes et al. [37]	2021	Genetic association study	Genetics	-
Rødevand et al. [38]	2021	Genetic association study	Genetics	-
Picco et al. [39]	2016	Cross-sectional study	Genetics	6616
Prieto et al. [40]	2016	Cross-sectional study	Genetics	7316
Dow et al. [41]	2024	Experimental study	Genetics	-
Zheng et al. [42]	2024	Bioinformatics analysis	Genetics	-
Zhang et al. [43]	2021	Genetic analysis	Genetics	-

Cardiovascular Dysregulation

Table 2 shows the pertinent findings of articles that analyze the relationship between cardiovascular dysregulation and BD pathophysiology.

**Table 2:** Pertinent findings of studies describing cardiovascular dysregulation as mechanism of BD pathophysiology BD: bipolar disorder, MI: myocardial infarction, MRI: magnetic resonance imaging, HPA: hypothalamic-pituitary-adrenal Source: [13-20]

Study	Pertinent findings
Chen et al. [13]	Hypertension is the most contributive physical illness to sudden cardiac death in BD patients. BD patients older than 50 were found to have additional structural cardiac changes that put them at increased risk for sudden cardiac death.
Prieto et al. [14]	This study assessed 334 individuals with BD type 1 for seven years in a US town and found that increased psychosis was a risk factor for non-fatal MI or stroke.
McGowan et al. [15]	BD is associated with a wider pulse pressure, which is suggested to be a subclinical indicator for CVD as widened pulse pressures are associated with arterial wall stiffness.
Najar et al. [16]	A seven-year longitudinal study assessed cardiometabolic risk indicators (CMRIs) and found that risk factors for hypertension and dyslipidemia that were elevated at baseline and then increased in patients with BD compared to healthy controls. A sensitivity analysis was performed to exclude participants on treatment for hypertension or dyslipidemia that could affect their CMRI.
Wageck et al. [17]	This study measured Coronary Calcium Scores (CCS) as a non-invasive measure of coronary atherosclerosis in BD patients hospitalized in the psychiatric ward. Their methods for a positive CCS were greater than the 75th percentile in calcium score. This study determined that an increased number of hospitalizations was associated with a higher CCS and was more likely to result in myocardial infarction.
Chen et al. [18]	This study assessed the Left Ventricular Mass Index (LVMI) in patients with BD and found it was positively correlated with inflammatory markers. They had an incidental finding of increased LVMI in male BD patients that participated in this study, which is significantly correlated with the length of time the patient has had BD.
Nunes et al. [19]	Lipid levels of those with and without mood disorders was assessed, and the controls and mood disorder participants were further separated by tobacco use disorder status.The calculated atherogenic index of plasma and atherogenic coefficient were elevated in BD patients.
Knedeisen et al. [20]	Using MRI to assess epicardial fat tissue (EAT) and adrenal gland (AG) volumes to determine HPA axis dysregulation in BD and major depressive disorder (MDD) patients and its effect on cardiovascular mortality. A 10-year cardiovascular mortality risk was calculated, and the data were compared between patients who had MDD and bipolar disorder. This study found EAT and AG volumes to be greatly increased in the bipolar disorder participants, indicating increased HPA axis dysregulation in BD patients. This links increased chronic stress and cortisol from HPA axis dysregulation to EAT and increased cardiometabolic risk with BD.

Data extracted from studies that are part of this review identify pathways of cardiac dysregulation that correlate with BD, leading to a possible pathophysiology that contributes to CVD [13,14,15,16,17,18 ]. Among the most significant factors identified is blood pressure dysregulation [13,14,15,16]. One study found that in BD patients younger than 50, hypertension (HTN) and venous disorders increase the risk of sudden cardiac death [13]. Furthermore, a seven-year longitudinal study determined that patients with BD exhibited both a higher baseline and increased progression of hypertension and dyslipidemia compared to their peers [14]. Beyond hypertension, BD patients were found to have a widened pulse pressure compared to their healthy peers, suggesting a subclinical indicator for cardiovascular disease [15]. 

A study assessing dyslipidemia in BD patients used the Atherogenic Index of Plasma (AIP) and atherogenic coefficient (AC) to measure lipid ratios that are associated with increased atherogenesis. This study determined that both AIP and AC were increased in BD patients along with classic atherogenic markers, and a possible physiologic mechanism is inflammatory, oxidative, and nitrosative stress pathways [19].

Studies analyzing structural aspects of cardiac dysregulation found that an increased number of psychiatric hospitalizations was associated with coronary calcium scores (CCS) greater than the 75th percentile [18]. An elevated CCS is a known indicator of coronary atherosclerosis [18]. In addition, research conducted suggests that the presence of psychosis in BD patients may increase their risk for non-fatal myocardial infarction or stroke [14]. Furthermore, patients with longer duration of BD present with increased left ventricular mass index (LVMI), which has been shown to be associated with increased levels of inflammatory markers such as platelet-lymphocyte ratio (PLR), C-reactive protein (CRP), and serum triglycerides [18]. Another study indicates that BD patients older than 50 had significant structural heart damage from conditions such as congestive heart failure (CHF) and HTN, increasing their risk for sudden cardiac death [13]. Further studies assessed epicardial fat with adrenal gland volume as a proxy for the hypothalamus-pituitary-adrenal axis dysregulation as a potential mechanism for CVD. Their results show increased epicardial fat and adrenal gland volumes in BD patients [20]. These findings indicate a physical change in cardiovascular function associated with BD.

Endothelial Dysfunction

Table 3 shows the pertinent findings of articles that analyze the relationship between endothelial dysfunction and BD pathophysiology.

**Table 3:** Pertinent findings of studies describing endothelial dysfunction as the mechanism of BD pathophysiology BD: bipolar disorder, cEPC: circulating levels of the endothelial progenitor cell, RHI: reactive hyperemia index, NO: nitric oxide, BDNF: brain-derived neurotrophic factor Source: [21-25]

Study	Pertinent findings
Goldstein et al. [21]	Microvacular reactivity changes were found in BD patients aged 13-20 years old. Coronary microvascular dysfunction starts to occur before gross structural changes to the heart are visible in BD patients.
Kennedy et al. [22]	This study assessed endothelial health in BD patients with depression vs mixed/hypomanic states. Endothelial function was weaker in the BD-depression group compared to the healthy control group. Endothelial health was increased in the BD-mixed/hypomanic group compared to the control group.
Liou et al. [23]	Lower endothelial function, measured using cEPCs, correlated with more severe manic and psychotic symptoms. Levels of cEPC were not significantly different between overall BD patients and healthy patients.
Kennedy et al. [24]	Lower brain volumes in young BD patients is associated with high endothelial function, measured by RHI. This may be due to NO and BDNF elevation during synptic pruning. These two molecules are involved with endothelial function
Barbosa et al. [25]	Klotho, an anti-aging protein and inducer of NO, is elevated in BD patients. This may be the result of a compensatory mechanism in endothelial distress.

Endothelial dysfunction with BD is a well-established connection [21]. Multiple studies support this by using the Reactive Hyperemia Index (RHI) as a measure of endothelial function, which is often associated with inflammation [21,22,23,24,25]. In fact, elevated RHI was found to be associated with increased severity of mania in BD, while a lower RHI was found in participants with depressive BD. Further studies examined correlations between RHI and brain anatomy, particularly in regions regulating mood stability [23]. Authors of this study found that higher RHI was associated with smaller cortical thickness in the temporal lobe and insular cortex in BD and control patients, but postulate that BD patients might be more sensitive to this association. This study discusses the physiologic mechanisms that regulate endothelial function, like nitric oxide (NO) and brain-derived neurotrophic factor (BDNF), which also regulate synaptic pruning [24]. Similarly, Barbosa et al. (2017) highlighted the role of a protein known as Klotho, which is associated with increased nitric oxide and subsequent endothelial function, and is found to be increased in BD patients [25]. 

Another measure of endothelial dysfunction is through circulating levels of endothelial progenitor cells (cEPCs), which are elevated in patients with manic symptoms. This association is independent of other cardiovascular risk factors and provides a possible mechanism for the increased risk of CVD in BD patients [23]. Furthermore, one study looks at coronary microvascular reactivity (CMVR), a metric to determine cardiovascular impairment. CMVR is significantly lower in BD participants, providing early evidence of CVD and suggesting an inflammatory mechanism [21].

*Inflammation* Table 4 shows the pertinent findings of articles that analyze the relationship between inflammation and BD pathophysiology.

**Table 4:** Pertinent findings of studies describing inflammation as the mechanism of BD pathophysiology BD: bipolar disorder, CRP: C-reactive protein, GDF-15: growth differentiation factor-15, CVD: cardiovascular disease Source: [26-33]

Study	Pertinent findings
Stubbs et al. [26]	Association between elevated CRP and sedentary levels in BD patients
Prins et al. [27]	Elevated CRP levels in BD, establishing an inflammatory state
Oral et al. [28]	Elevated levels of endothelial markers in BD patients
Turan et al. [29]	Higher levels of cell adhesion molecules and E-selectin seen in BD patients, especially during manic episodes
Tsai et al. [30]	BD patients exhibited increased left ventricular hypertrophy and hyperdynamic heart function, associated with elevated inflammatory markers
Tsai et al. [31]	Systemic inflammation cytokines seen in high concentrations in BD patients
Yang et al. [32]	Elevated GDF-15 in BD patiens
Rødevand et al. [33]	Shared genetic loci between BD and CVD

Several inflammatory markers have been identified in individuals with BD that may contribute to increased cardiovascular risk [26,27,28,29]. CRP, a known marker of systemic inflammation, has been found to be elevated in BD and is associated with cardiovascular outcomes. In addition, higher sedentary behavior in BD patients has been linked to elevated CRP levels [26,27]. Markers of endothelial dysfunction, including endocan and urotensin-II (U-II), have been reported to be significantly higher in BD patients compared to controls, particularly in manic or depressive episodes [28]. Elevated levels of intracellular adhesion molecule-1 (ICAM-1), vascular cell adhesion molecule-1 (VCAM-1), and E-selectin have also been found in BD patients, with higher levels observed during manic episodes [29].

Cytokines involved in systemic inflammation, including tumor necrosis factor-alpha (TNF-α), interleukin-6 (IL-6), and interleukin-8 (IL-8), have been measured at higher concentrations in BD patients [30,31]. Notably, IL-8 was associated with increased left ventricular hypertrophy (LVH), while soluble TNF receptor 1 (sTNF-R1) correlated with left ventricular function [30]. In addition, leukocyte count and systemic inflammatory markers were significantly higher in BD patients who experienced early cardiovascular mortality [31].

Further evidence of inflammatory activation in BD includes the elevation of growth differentiation factor-15 (GDF-15), a marker associated with aging and cardiovascular risk, with levels found to be significantly higher in BD patients compared to controls [32]. Genetic studies have additionally identified shared genetic loci between BD and cardiovascular disease, particularly in pathways related to immune system regulation and inflammatory signaling [33].

Oxidative Stress and Metabolic Dysregulation

Table 5 shows the pertinent findings of articles that analyze the relationship between oxidative stress and metabolic dysregulation and BD pathophysiology.

**Table 5:** Pertinent findings of studies describing oxidative stress and metabolic dysregulation as mechanism of BD pathophysiology BD: bipolar disorder, DNA: deoxyribonucleic acid, RNA: ribonucleic acid, HRV: heart rate variability, PWV: pulse wave velocity, CVD: cardiovascular disease Source: [34-36]

Study	Pertinent findings
Hatch et al. [34]	Elevated lipid hydroperoxides and protein carbonylation associated with increased vascular dysfunction in BD
Coello et al. [35]	Genetics of BD showed high DNA and RNA oxidation, as well as a potential familial trait.
Xie et al. [36]	Cardiac autonomic dysfunction in BD, such as lower HRV and increased PWV, may be an indicator of increased CVD risk.

Evidence suggests that oxidative stress plays a critical role in the cardiovascular dysfunction observed in BD [34,35,36]. Elevated levels of oxidative stress markers, including lipid hydroperoxides (LPH) and protein carbonylation (PC), have been associated with increased vascular dysfunction in BD [34]. Among adolescents with BD, higher LPH levels correlated with increased pulse pressure and greater carotid intima-media thickness (cIMT), a proxy measure of atherosclerosis [34]. Similarly, oxidative stress-induced DNA and RNA damage is found to be significantly higher in young BD patients and their unaffected relatives compared to healthy controls, suggesting that increased oxidative stress may be a trait phenomenon associated with familial cardiovascular risk. BD patients show 21.8% higher DNA damage and 14.8% higher RNA damage, while relatives exhibited similar elevations despite being unaffected [35].

Beyond oxidative stress, autonomic and metabolic dysregulation have been observed in BD, further contributing to cardiovascular abnormalities. A study comparing patients with major depressive disorder (MDD) and BD found that BD patients exhibited significantly lower heart rate variability (HRV) and increased pulse wave velocity (PWV), indicating heightened arterial stiffness and autonomic imbalance [36].

*Genetics* Table 6 shows the pertinent findings of articles that analyze the relationship between genetics and BD pathophysiology.

**Table 6:** Pertinent findings of studies describing genetics as the mechanism of BD pathophysiology CVD: cardiovascular disease, BD: bipolar disorder, SNP: single-nucleotide polymorphism Source: [37-43]

Study	Pertinent findings
Fürtjes et al. [37]	Shared genetic etiology persisted between bipolar disorder and coronary artery disease.
Rødevand et al. [38]	One hundred twenty-nine shared loci were identified between bipolar disorder and CVD risk factors, suggesting mixed genetic effects.
Picco et al. [39]	Demonstrated a higher prevalence of CVD in BD patients of Indian origin as opposed to those of Chinese and Malay ethnic roots,
Prieto et al. [40]	Strong association was observed between the rs476593 section on the CACNA1C gene and the phenotype of cardiac dysrhythmias.
Dow et al. [41]	Action potential propagation velocity was slowest in the bipolar patient cell line carrying the CACNA1C SNP–associated genetic risk factor.
Zheng et al. [42]	Two key hub genes (CX3CR1 and ST6GAL1) were identified as biomarkers for arteriosclerosis in bipolar disorder patients.
Zhang et al. [43]	Neuroticism was causally linked to increased risk of BD and CVD through shared genetic mechanisms, identifying key genes like MAD1L1 and ARNTL.

Multiple studies as part of this review identify genetic mechanisms as a possible pathophysiologic correlator between BD and CVD [37,38,39,40,41,42,43]. A partial genetic overlap exists between the BD and CVD phenotypes, with one study identifying up to 129 shared loci between the two conditions [37,38]. Another study identified a higher prevalence of CVD in BD patients of Indian origin as opposed to those of Chinese and Malay ethnic roots, thereby drawing light to a role that genetics might play in cases of ethnic differences [39].

**Figure 2:**
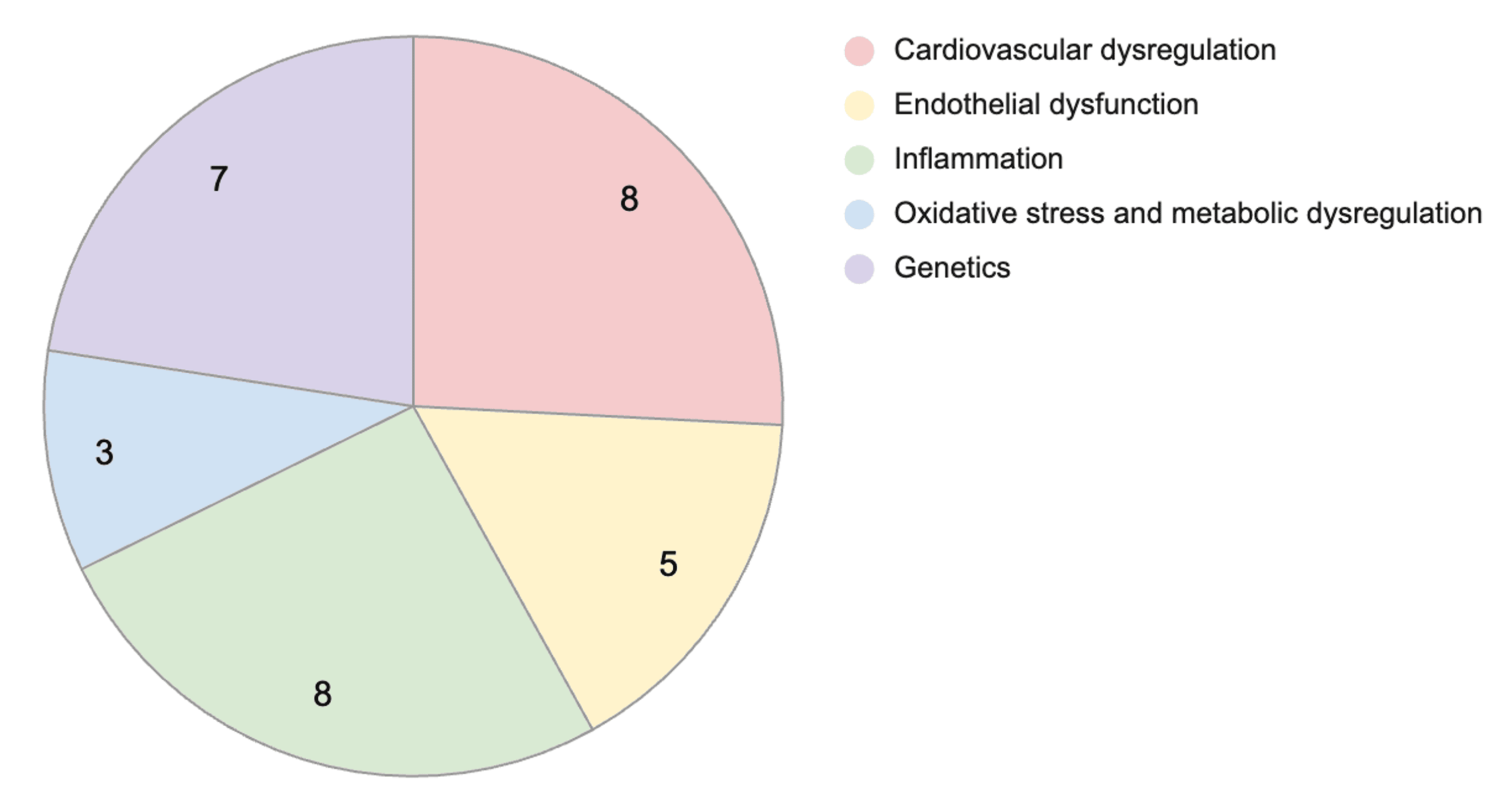
Pie chart of CVD pathophysiologic mechanisms in BD patients This pie chart summarizes the findings of the studies recorded in Table 1 and groups the pathophysiologic mechanisms of CVD in BD patients based on the number of papers identified in this review. Source: [13-43]. Image credits: First author (D. Ram).

Studies have even gone on to identify specific genes that may be involved in increasing CVD risk in BD patients. CACNA1C genetic mutations with single-nucleotide polymorphisms have been identified to cause fatal arrhythmias, cardiomyopathy, prolonged QTc syndrome, and sudden cardiac death in BD patients [40,41]. CX3CR1 and ST6GAL1 genes have been identified in association with atherosclerosis [42]. Other genes that have been identified as key genetic hubs include MAD1L1 and ARNTL [43]. Figure 2 shows a pie chart of the most prevalent evidence-based pathophysiologic mechanisms of CVD in BD patients based on the number of papers found in this systematic narrative review of recent literature: cardiovascular dysregulation with eight articles, endothelial dysfunction with five articles, inflammation with eight articles, oxidative stress and metabolic dysregulation with three, and genetics with seven articles.

Discussion

The findings of this systematic review highlight the multifactorial nature in which CVD develops in BD individuals, suggesting the convergence of physiologic, molecular, and genetic factors. CVD continues to remain one of the leading causes of morbidity and mortality in BD patients, and it is vital that healthcare providers understand what predisposes BD patients to cardiovascular risk to help improve their health outcomes and quality of life. This review identifies five interrelated mechanisms that contribute to CVD in individuals with BD: cardiovascular dysregulation, endothelial dysfunction, inflammation, oxidative stress and metabolic regulation, and genetics. By examining these processes along a continuum from a broader structural level to a more microcellular and molecular level, this review further explores the complex interplay between these mechanisms.

At a social level, male patients with BD have an increased cardiovascular burden and risk compared to females [44]. This review depicts that LVMI is increased in both male and female patients with BD due to the role of inflammatory markers. However, in males specifically, a longer duration of BD correlates with an elevated LVMI [18]. Increased LVMI is due to adipose tissue dysfunction and inflammation in BD patients, leading to structural cardiac remodeling through multiple cycles of tissue destruction and repair. Through these cycles, the myocardium hypertrophies and elevates LVMI, which is a significant risk factor in the development of congestive heart failure [45]. This review hypothesizes that the correlation between increased LVMI and males with BD is because men lack estrogen and its subsequent protective effects. Estrogen has been found to inhibit calcineurin activity. Calcineurin is an important protein that has functions in the development of cardiac musculature and vessels. Therefore, the lack of calcineurin inhibition by decreased estrogen levels leads to increased cardiac remodelling and LVMI in males [46,47]. Thus, by establishing BD as an inflammatory state, this paper identifies a possible pathogenesis that contributes to the comorbid cardiovascular burden that BD patients suffer.

Aside from hormonal and metabolic control, some genes appear to play a role in increasing the cardiovascular burden at a structural level. Polymorphism of CACNA1C, a gene prevalent in BD populations, is shown to be co-occurring in those patients who develop CVD [40,41]. CACNA1C is a gene that codes for the α1C subunit of L-type calcium channels (LTCCs) that regulate excitation-contraction coupling of cardiac myocytes. Single-nucleotide polymorphisms of this gene thereby result in decreased function of these vital calcium channels [48,49]. Furthermore, mutations to MAD1L1, a checkpoint gene, result in issues related to cell division [50]. While its role is more implicated in the incidence of lung cancer, it can be hypothesized that uninterrupted cellular division of cardiac myocytes can result in reduced cardiac function in patients with BD. 

An effect of these structural changes is blood pressure dysregulation, with one study proposing that higher blood pressure in those with BD contributed to structural changes in the heart. The widened pulse pressures in BD patients contribute to overall arterial wall stiffness over time, which this review hypothesizes is due to fluctuating moods that characterize this condition [13]. Further papers discuss how emotional stressors can lead to increased blood pressure, and chronic stressors could modify neuronal pathways that control the sympathetic nervous system [51]. In a chronic mood disorder like BD, states of mania can be seen as emotional stressors that this review suggests could explain its effects on widened pulse pressures, which further contribute to CVD risk. Another hypothesis is that in a manic state, increased catecholamines like norepinephrine and serotonin are released, which is seen in the pathogenesis of essential hypertension and BD, thus contributing to widened pulse pressures [52]. 

Furthermore, a seven-year study determined that patients with BD exhibited a higher baseline of blood pressure and increased progression to hypertension and dyslipidemia compared to healthy controls [15]. Based on the findings of this systematic review, the relationship between dyslipidemia and hypertension in BD patients is cyclical in nature. Hypertension leads to increased shear stress on arterial walls, causing an upregulation of lipid oxidation enzymes and increased activation of the angiotensin type 1 receptor. This leads to augmented lipid uptake by the vessel walls, further contributing to dyslipidemia. In addition, dyslipidemia in BD patients can lead to plaque deposition in the arteries and further stenosis, resulting in subsequent elevations in blood pressure [53]. Thus, not only are key CVD risk factors increased in BD patients, but the progression of dyslipidemia can cause structural changes to the heart as well.

Complementing the dyslipidemia findings, emerging data also point to oxidative stress and metabolic imbalance as key pathophysiologic pathways linking BD with early CVD. Elevated LPH and PC - well-established markers of oxidative stress - have been found to correlate with carotid intima-media thickness in adolescents with BD, suggesting that oxidative injury to lipids and proteins may contribute to vascular dysfunction from an early stage [34]. Coello et al. (2024) further identified increased oxidative damage to DNA and RNA in both young BD patients and their unaffected first-degree relatives, supporting the hypothesis that oxidative stress may represent a trait vulnerability with familial underpinnings [35]. 

Metabolic dysregulation is also evident, with decreased HRV and increased PWV in BD patients indicating impaired autonomic function and increased arterial stiffness compared to individuals with unipolar depression [36]. These findings are consistent with a broader model of accelerated cellular aging in BD, wherein redox imbalance, mitochondrial dysfunction, and metabolic shifts interact with inflammation to promote early-onset cardiovascular pathology. Furthermore, the gene ARNTL plays an integral part in lipid metabolism by influencing lipogenesis in adipocytes in a circadian fashion. Its role in fat metabolism, therefore, predisposes BD patients to increased risk of hypertension, cardiomyopathy, and metabolic syndrome [54]. The convergence of these mechanisms highlights the urgency of early detection and intervention strategies targeting oxidative and metabolic pathways in BD populations at risk for CVD.

Furthermore, several social factors play a key role in the development of atherosclerosis in BD patients. When determining structural changes of cardiac dysregulation, studies found that CCS, a marker of atherosclerosis, above the 75th percentile was associated with increased psychiatric hospitalizations. A higher CCS is related to coronary atherosclerosis, which is more likely to result in MI. A few papers hypothesize that due to BD patients' episodes of mania and depression, they are prone to making poorer lifestyle choices. A few examples of these lifestyle choices include increased occurrences of substance use, gambling, and eating foods high in fats [55]. These lifestyle choices are in line with key characteristics of BD, such as impulsivity, leading us to understand how lowered inhibitions can cause BD patients to make poorer lifestyle choices during their manic phases [56]. Many of these lifestyle choices lead to increased atherogenesis, thus explaining the subsequent elevation in CCS seen in this review. Given this possible association between mood instability and cardiac dysfunction, healthcare providers should take care to counsel patients with BD on how to make healthier lifestyle choices. 

At the genetic level, the CX3CR1 gene has been shown to be associated with atherosclerosis in BD patients via vascular inflammation injury. The CX3CR1 gene, which is activated by inflammatory agents such as TNF-α and LPS, mediates the interaction between inflammatory and vascular cells, thereby promoting plaque development [42]. Another gene associated with atherosclerosis development in BD patients is ST6GAL1. This gene is anti-inflammatory in nature and is found in lower levels in BD patients [42]. While its exact role in the development of atherosclerosis is not fully understood, it can be hypothesized that its low levels in BD patients encourage a prolonged inflammatory state, thereby promoting plaque development in a similar manner to the CX3CR1 gene.

In addition to genetic predispositions, physiological changes can contribute to the cardiovascular risk in BD. Dysfunction at the level of the vascular endothelium is a key driver of CVD risk in BD patients. Blood vessels are lined with endothelial cells, which directly function in regulating blood flow and maintaining vascular health [57]. Dysfunction of these cells leads to the development of atherosclerosis, the main mechanism of cardiovascular diseases. Research using circulating endothelial progenitor cells (cEPCs) has elucidated a relationship between endothelial health and mood state in BD. Lower levels of cEPCs were correlated with more severe manic and psychotic symptoms. Our analysis implies this may result from the oxidative stress and systemic inflammation that can occur during severe mood episodes, leading to endothelial injury reflected by decreased cEPCs. In addition, the hypothalamic-pituitary-adrenal axis may be dysregulated in severe mood states, leading to high cortisol levels and impaired vascular regeneration. However, there was no significant difference in cEPC simply between BD patients and healthy patients [23]. This result suggests that poor endothelial function in mood disorders may come into play only as the disorder becomes more severe, rather than at baseline. This review suggests that as the mood state becomes more severe and vascular health is compromised, CVD risk increases in BD patients. 

Kennedy et al. found that youth in the BD-depressed category had significantly lower RHIs than in the healthy control group, suggesting poorer endothelial function during depression [24]. One explanation for this is the elevated inflammatory state associated with depression and reduced endothelial responsiveness from a possible change in vascular signaling molecules [58]. Healthy controls however had significantly lower RHIs than the BD-mixed/hypomanic category [22]. This suggests that poor endothelial function is associated with depressive symptoms in BD patients and increases CVD risk. High stress manic or hypomanic states have elevated circulating catecholamines and sympathetic activity, which may augment vascular signaling leading to enhanced endothelial responsiveness and RHI. Vascular tone may also be elevated during manic symptoms, leading to increased endothelial responsiveness. These results suggest that endothelial responsiveness may be deceptively increased in mania due to sympathetic activity, while CVD risk remains high. There was an elevated RHI with manic symptoms and a low RHI with depressed symptoms, suggesting that BD may have an overall RHI that is close to normal levels because the two extreme symptoms balance each other’s effect. Significant differences were seen between subgroups of BD patients and healthy patients; however, the direction of this trend requires further study. 

Vascular and endothelial health has an evident role in the risk of CVD in BD patients. To further understand this relationship, we must examine the smaller molecular mediators of the process. Nitric oxide (NO) and BDNF are two such molecules that may drive the effects on cardiovascular health in BD pathology. Young patients exhibited a trend of lower brain volumes having higher RHI, suggesting that synaptic pruning may be involved [24]. NO is known to promote synaptic pruning and endothelial function [59]. BDNF is also involved in synaptic pruning and has an important role in endothelial health and neurogenesis [24]. The role of these two molecules provides a basis for the association between higher RHI and lower brain volume. 

Vascular endothelial dysfunction has been shown to occur in 13-20-year-old patients, even without large-scale cardiac left ventricular changes. This provides evidence that vascular disruption can be present in young BD patients well before the expected onset of traditional cardiovascular symptoms [22]. Our hypothesis for this is that endothelial cells are very susceptible to early systemic insults, including inflammation and oxidative stress. These early changes can impact vascular tone and reactivity before the effects lead to structural remodeling of the heart. In young patients, this effect is likely related to increased levels of NO and BDNF, which may be elevated due to synaptic pruning. Pruning within the areas of the brain responsible for emotional regulation may further dysregulate mood in BD patients. Changes in vascular endothelium may present a clinically significant opportunity for early detection, prevention, and management of CVD. 

Another study demonstrated that Klotho, an anti-aging and antioxidant protein, is decreased in healthy patients compared to BD patients [25]. Klotho is also known as an inducer of NO, which plays an important role in vasodilation and acts as a link between Klotho and endothelial health. Through this link, Klotho’s association with the neuropsychiatric features of BD suggests that vascular health is critical for well-being in BD patients. Given its antioxidant and anti-aging properties, it was expected that lower levels of Klotho would be seen in BD patients; however, results demonstrated the opposite trend. An explanation for this is that early phases of BD may have more rampant pathological processes at work, and as the disease progresses, there is a reactionary increase in Klotho [25]. Physiological stress may lead to an elevation of Klotho as an adaptive attempt to mitigate endothelial injury and preserve vascular health. The consistent trends in Klotho levels across BD patients further corroborate that endothelial dysfunction could be a marker of disease progression. 

Molecular mediators such as NO, BDNF, and Klotho provide a link between BD and vascular health, while also revealing how vascular changes may emerge early in the disease course. A closer examination reveals that inflammation plays a complementary role in this process, creating an additional connection between BD and cardiovascular pathology. Elevated levels of inflammatory biomarkers, including CRP, IL-6, IL-8, and TNF-α, have been repeatedly observed in BD cohorts and are closely associated with subclinical cardiac abnormalities such as left ventricular hypertrophy and hyperdynamic function [30]. These biomarkers play central roles in orchestrating the inflammatory cascade: CRP is an acute-phase reactant produced by the liver in response to systemic inflammation, while IL-6, IL-8, and TNF-α are pro-inflammatory cytokines that drive immune cell recruitment, endothelial activation, and vascular injury [27,30].

Moreover, BD is known to be a pro-inflammatory state, which is exacerbated by manic and depressive episodes that can contribute to increased endothelial dysfunction and elevated CCS [17]. This inflammatory state is thought to occur due to the frequency of manic and depressive episodes, causing a cumulative inflammatory burden on BD patients over the duration of their illness, as evidenced by elevated inflammatory markers [60].

Endothelial activation markers such as ICAM-1, VCAM-1, and E-selectin further suggest ongoing vascular injury during acute mood episodes [29], while increased levels of endocan and urotensin-II reflect persistent endothelial dysfunction beyond the acute phases of illness [28]. Notably, although inflammatory markers tend to peak during manic and depressive episodes, several studies report that elevated inflammation persists during euthymic periods, suggesting that chronic low-grade immune activation may underlie the progressive vascular damage in BD [29,30]. Although chronic inflammation in BD is well established, it remains unclear to what extent the immune dysregulation is driven by genetic versus environmental factors. Prins et al. (2016) demonstrated a causal association between genetically elevated CRP levels and cardiovascular outcomes, strengthening the biological plausibility of this link [27]. Meanwhile, Stubbs et al. (2015) identified sedentary behavior in BD patients as a behavioral contributor to CRP elevation [26]. Given the consistent association between inflammatory markers and cardiovascular outcomes, these biomarkers may hold promise for early cardiovascular risk stratification and preventative interventions in young individuals with BD. Together, these findings implicate both intrinsic immune dysregulation and modifiable lifestyle factors in the inflammatory processes that underpin premature cardiovascular pathology in BD. However, the interpretation of inflammatory marker data in BD is limited by the heterogeneity of study designs, variability in patient mood states at the time of sampling, and the potential confounding effects of psychotropic medications.

The pathology of focus in this review is BD; however, depression and cardiovascular disease are closely linked as well, with compelling evidence suggesting a bidirectional relationship that significantly impacts patient outcomes. As described by Nemeroff and Goldschmidt-Clermont in their comprehensive review, depression not only serves as a risk factor for the development of cardiovascular disease, but it also worsens prognosis in patients with existing heart conditions [61]. The authors highlight the complex interplay of biological and behavioral mechanisms, including autonomic dysregulation, increased inflammatory markers, platelet activation, and poor health behaviors, that contribute to this connection. Recognizing and treating depression in patients with cardiovascular disease is therefore essential for improving both psychological and cardiac outcomes.

While this review focuses primarily on pathophysiologic mechanisms, it is also important to consider emerging interventions that may mitigate cardiovascular risk in BD. Given the role of systemic inflammation and stress-related pathways, several psychosocial interventions, including mindfulness-based stress reduction, cognitive behavioral therapy (CBT), and structured physical activity, have demonstrated reductions in inflammatory markers such as CRP and IL-6 in mood disorder populations. These strategies may improve autonomic function, reduce endothelial stress, and serve as non-pharmacological avenues to lower cardiovascular burden. Although evidence directly linking these interventions to improved cardiac outcomes in BD remains limited, studies in patients with depression have shown promising results. For instance, Carmin et al. (2024) reported that pharmacotherapy and psychotherapeutic mental health treatments appear essential for reducing hospitalizations and ED visits, highlighting the potential benefits of addressing mood symptoms as part of an integrated care model [62]. Future studies should explore whether these interventions translate to cardiovascular benefit in BD, particularly in high-risk subgroups.

Limitations

As a systematic narrative review, our study carries limitations. A few limitations of this review include limited research regarding elevated CCS scores in the BD patient population, as the study reported in this review only looked at a population admitted to a psychiatric ward. Further research on CCS scores in BD patients without severe illness should be conducted to better extrapolate findings that can apply to the general populace. Another limitation is the lack of investigation on the cardiovascular implications of estrogen in BD females and estrogen’s relation to elevated LVMI scores. While the studies used in this review differentiated between male and female patients, there was a lack of focus on female BD patients. This review faced limitations when looking at the genetic components involved in CVD development in BD patients due to the novelty of gene therapy research. This review however identifies gene therapy as a field of medicine that requires further study to elucidate the new pathogenesis of CVD in the BD population. This review also found that further study is needed in using endothelial function as a screening approach for CVD in BD patients. In addition, the interpretation of inflammatory markers in BD is limited by the heterogeneity of the study design, mood states at the time of sampling, and the potential confounding effects of psychotropic medications. Furthermore, as a narrative review, no statistical analysis was performed by the reviewers. This emphasizes the need for potential meta-analysis to be performed by specialists in the field for accurate quantitative data analysis. Finally, this paper also faced limitations in accessing certain articles that are behind paywalls or require translation from other languages to access.

## Conclusions

The findings of this review thus highlight the possible pathophysiological mechanisms that predispose BD patients to early-onset CVD, which continues to be a leading cause of mortality in this population. Given the rising global prevalence of BD, it is vital that physicians and other healthcare providers understand the biosocial factors that contribute to the cardiovascular comorbidities of their patients, to best educate and support them. Genetic studies and early cardiac interventions may help BD patients lower their risk of concurrent CVD.
